# Vesicovaginal and vesicorectal fistula in a patient with systemic sclerosis: A case report

**DOI:** 10.1002/ccr3.8550

**Published:** 2024-02-22

**Authors:** Mohammad Quteineh, Sajedah N. Obeid, Khayry Al‐Shami, Hamdah Hanifa

**Affiliations:** ^1^ Department of Clinical Medical Sciences, Faculty of Medicine Yarmouk University Irbid Jordan; ^2^ Faculty of Medicine University of Kalamoon Al‐Nabk Syria; ^3^ Al‐Dandashi National Group Yaafour Damascus Syria

**Keywords:** autoimmune disease, case report, fistula development, scleroderma, systemic sclerosis, vesicorectal, vesicovaginal

## Abstract

**Key Clinical Message:**

This case highlights the need for further research to explore a potential link between systemic sclerosis and fistula development, and the importance of raising awareness among clinicians about this possibility for timely appropriate management.

**Abstract:**

The primary diagnosis in this case is systemic sclerosis (scleroderma) in a 47‐year‐old female patient with various clinical manifestations, including vesicovaginal and vesicorectal fistulas. The medical interventions and diagnostic workup involved an electrocardiogram, cardiac echocardiogram, pulmonary function tests, beta blockers, aspirin, inhaled corticosteroids, albuterol, endoscopy, biopsy, treatment for *Helicobacter pylori* infection, fluconazole for candida esophagitis, endoscopic dilation for achalasia, anticholinergic therapy for mixed urinary incontinence, gabapentin for neuropathic symptoms, analgesia for knee pain, and psychiatric treatment with selective serotonin reuptake inhibitors. The outcomes described in the case report include the diagnosis of systemic sclerosis, the identification of vesicovaginal and vesicorectal fistulas, the patient's medical history and symptoms over the years, and various treatments and management strategies.

## 1 INTRODUCTION

Systemic sclerosis, or scleroderma, is an immune‐mediated rheumatic disease characterized by fibrosis in the skin, internal organs, and vasculopathy.^1^ This rare condition, affecting approximately 1 in 10,000 people,^2^ has the highest mortality rate among all rheumatological diseases. Systemic sclerosis impacts multiple systems, including the respiratory, gastrointestinal, skin, and renal systems.^3^ Systemic sclerosis, commonly referred to as scleroderma, often presents with various co‐occurring disorders and complications. These include Raynaud's phenomenon, characterized by color changes in fingers or toes in response to cold or stress, interstitial lung disease leading to pulmonary fibrosis, gastrointestinal issues like gastroesophageal reflux disease (GERD), cardiac complications such as pericarditis and pulmonary hypertension, kidney involvement with scleroderma renal crisis (SRC), joint and muscle pain, Sjögren's syndrome resulting in dry eyes and mouth, pulmonary hypertension, skin ulcers, and peripheral neuropathy causing numbness and tingling in extremities. Effective management and monitoring of these concurrent conditions are crucial for enhancing the quality of life and overall health of individuals with systemic sclerosis.^4^

Respiratory manifestations, such as interstitial lung disease and pulmonary hypertension, have been reported in all variants of systemic sclerosis.^3^ Gastrointestinal involvement is observed in up to 90% of patients with esophageal symptoms, including heartburn, dysphagia, odynophagia, and regurgitation, being the most common.^5^ The skin often appears thickened, hardened, and tight in patients with systemic sclerosis. More extensive skin involvement is associated with more severe internal organ manifestations, poorer prognosis, and increased disability, particularly in the early phase of the diffuse cutaneous scleroderma subset.^6^

SRC is a feared complication of systemic sclerosis, presenting symptoms such as malaise, fatigue, headaches, seizures, fevers, encephalopathy, blurred vision, and dyspnea, along with accelerated hypertension. Pulmonary edema, arrhythmias, myocarditis, and pericarditis may also develop and indicate poor prognosis.^7^

## 2 CASE HISTORY/METHODS

A 47‐year‐old female with a medical history of systemic sclerosis and depression, both diagnosed in 2020, and multiple food allergies, was referred to a gastroenterology clinic by the gynecology department for evaluation of a suspected vesicorectal fistula. The patient reported urine leakage from the anus. Prior to 2015, the patient's condition had been stable, but she began experiencing shortness of breath and intermittent chest pain.

As part of her diagnostic workup, an electrocardiogram (ECG), cardiac echocardiogram (Echo), and pulmonary function tests (PFTs) were performed with no other pulmonary tests performed. The ECG revealed atrial fibrillation, while the Echo results were normal. Consequently, the patient was started on beta blockers and aspirin. The PFT indicated an obstructive pattern (Table 1), leading to the initiation of inhaled corticosteroids for two times a day and albuterol as needed. Despite this treatment, the patient experienced multiple exacerbations of her respiratory symptoms in the following years, resulting in two hospitalizations for symptom management.

**TABLE 1:** Pulmonary function test results for the patient.

FEV1	3.41	3.08
FVC	2.65	1.92
Ratio	62%	68%

Abbreviations: FEV1, forced expiratory volume in the first second; FVC, forced vital capacity.

The patient reported a long history of gastrointestinal symptoms that began in 2016, including dysphagia, bloating, and heartburn. Initially treated as a case of GERD and irritable bowel syndrome with omeprazole and dietary modifications, her symptoms persisted. An upper endoscopy and biopsy confirmed the presence of *Helicobacter pylori*, prompting the initiation of triple therapy. However, the patient's condition did not improve, and a subsequent endoscopy revealed candida esophagitis, which was treated with fluconazole, successfully alleviating her symptoms. In 2018, the patient experienced dysphagia once again, and manometry revealed type 1 achalasia, which was treated with endoscopic dilation.

**FIGURE 1:**
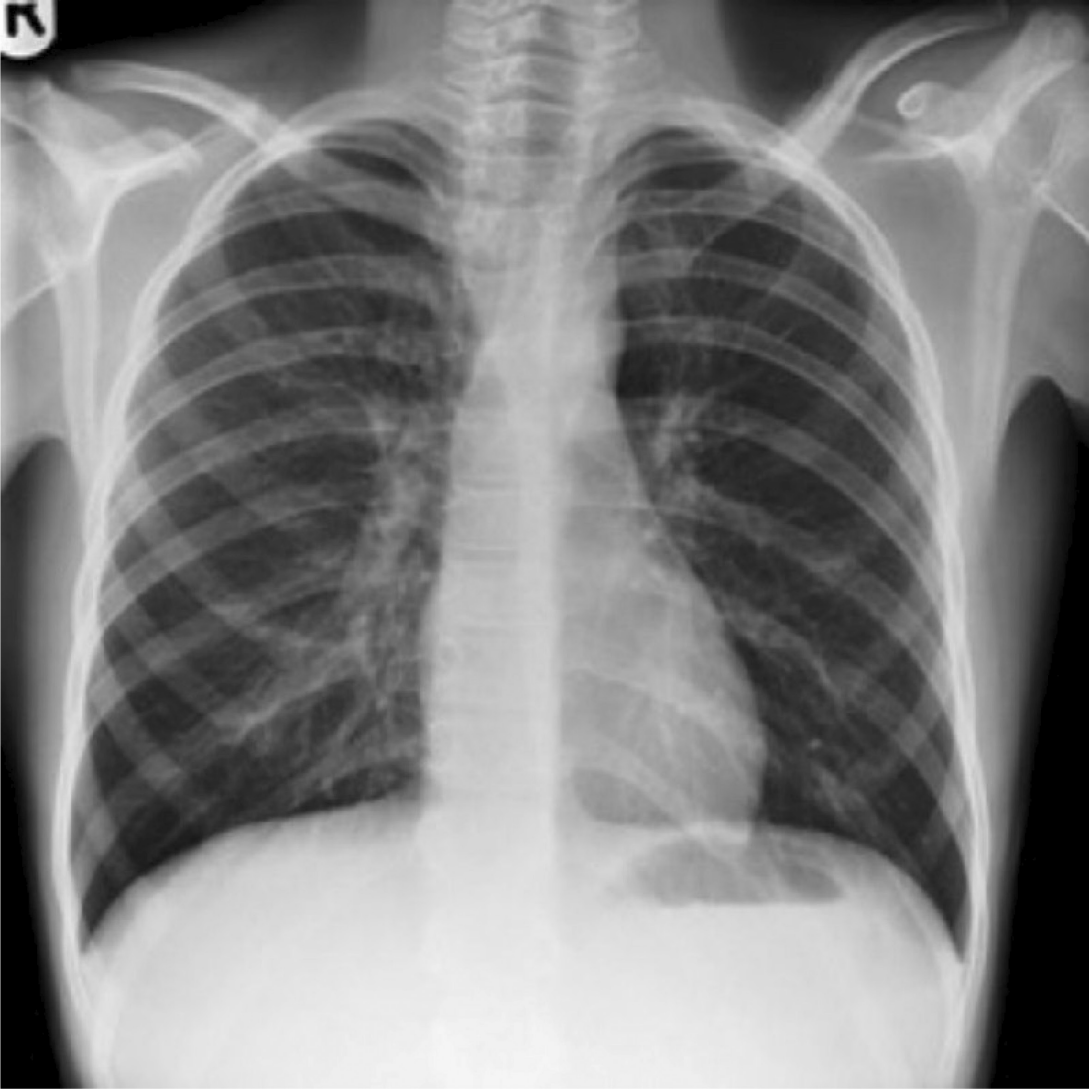
X‐ray picture showing left‐sided cervical rib.

In 2016, the patient also began experiencing urinary urgency, nocturia, and urinary incontinence. She reported urine leakage during coughing or laughing and episodes of incontinence associated with urgency. Consequently, a diagnosis of mixed urinary incontinence was made, and the patient was started on anticholinergic therapy, which resolved her symptoms. In 2017, the patient developed numbness and paresthesia in both upper limbs, particularly the left‐side fingers. A neck magnetic resonance imaging (MRI) scan showed no abnormalities, but neck X‐rays revealed a cervical rib (Figure 1). The patient was managed with gabapentin 300 mg orally twice a day.

**FIGURE 2:**
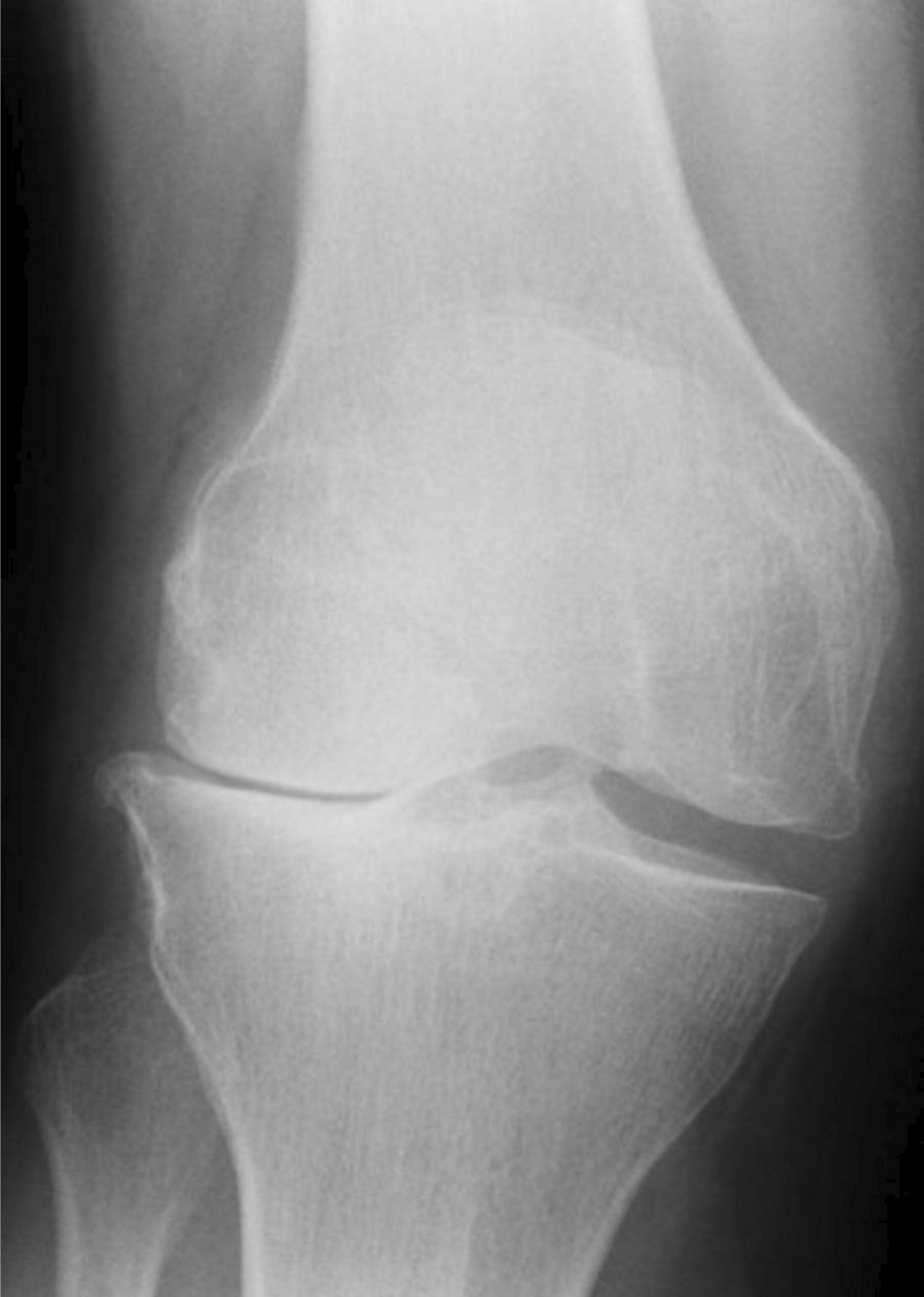
X‐ray picture showing severe right knee osteoarthritis.

The patient also experienced severe right‐sided knee pain that limited her mobility. Knee X‐rays revealed osteophytes and a loss of joint space, consistent with osteoarthritis (Figure 2), and she was managed with analgesia. In 2018, the patient started visiting a dermatology clinic due to a rash and itchiness throughout her body. A physical examination at that time identified erythematous papules behind both ears, occiput, and abdomen, leading to a diagnosis of Prurigo simplex.

In 2020, the patient consulted the rheumatology department, where multiple laboratory tests and rheumatological studies were conducted. Positive anticentromere antibodies led to a diagnosis of systemic sclerosis, in accordance with the American College of Rheumatology/European League (ACR/EULAR) against rheumatism criteria, and she was managed accordingly. Additionally, the patient was referred for psychiatric evaluation, as she exhibited signs of depression. She was diagnosed with major depressive disorder and began treatment with selective serotonin reuptake inhibitors.

The patient's most recent visit to the hospital involved a consultation at the gynecology clinic, where a pelvic examination confirmed the presence of urine in the vaginal vault. She was referred to the gastroenterology clinic for further evaluation, as she also reported urine leaking from her anus. A physical examination at the gastroenterology clinic revealed an obese female with a body mass index of 42, Raynaud's phenomenon, sclerodactyly in the fingers, and a blunted effect. A rectal examination did not detect a vesicorectal fistula. The patient underwent an MRI, which was inconclusive, but a subsequent cystoscopy confirmed the presence of a vesicorectal fistula.

The patient's family history was significant only for the early sudden cardiac death of her father at the age of 49; no family members had any rheumatological diseases. Her surgical history included an anterior cruciate ligament repair of her right knee 20 years prior and a mucosal resection for rhinosinusitis in 2016. The patient's current medications include methotrexate 10 mg, gabapentin 300 mg, fluoxetine 20 mg, aspirin 100 mg, bisoprolol 5 mg, and calcium and vitamin D supplements.

## 3 CONCLUSION AND RESULTS

In conclusion, we have presented a unique and complex case of a patient with systemic sclerosis who developed coexisting vesicorectal and vesicovaginal fistulas. Although there is no established association between systemic sclerosis and fistula development, this case warrants further investigation to explore a potential link. It is crucial to raise awareness among clinicians about the possibility of fistula development in systemic sclerosis patients, as this would ensure timely diagnosis and appropriate management.

Future studies could help elucidate the underlying mechanisms contributing to the development of fistulas in the context of systemic sclerosis and guide the development of effective prevention and treatment strategies. Understanding the relationship between these conditions may significantly improve patient outcomes and the overall management of systemic sclerosis and its potential complications.

## 4 DISCUSSION

Systemic sclerosis is a connective tissue disease that leads to skin and internal organ fibrosis.^1^ It is classified into several subgroups based on clinical presentation, with the most common being limited systemic sclerosis and diffuse systemic sclerosis.^2^ Common manifestations of systemic sclerosis include bilateral sclerodactyly, Raynaud's phenomenon, and severe digital ulceration. Diffuse systemic sclerosis has more widespread cutaneous involvement and systemic disease.^3^ Our patient was diagnosed with systemic sclerosis, which was complicated by GERD and pulmonary symptoms such as shortness of breath and joint discomfort. These features are typical in individuals with systemic sclerosis, but she also had vesicovaginal and vesicorectal fistulas, which led to urine leaking from the vagina and rectum. This is believed to be the first reported case of systemic sclerosis with vesicovaginal and vesicorectal fistulas worldwide. In this case, the patient with various symptoms and vesicorectal and vesicovaginal fistulas was diagnosed with systemic sclerosis using the ACR/EULAR criteria for systemic sclerosis.^8^

Gastrointestinal involvement has been observed in more than 90% of individuals with systemic sclerosis, while 74% had esophageal involvement. In a study of scleroderma patients, all individuals with erosive esophagitis demonstrated abnormal motility characterized by a lack of peristalsis in the distal esophagus. In other series, 50%–90% of systemic sclerosis patients had reflux esophagitis.^9^, ^10^ Reflux esophagitis can lead to a life‐threatening condition known as aortoesophageal fistula. Diffuse shallow ulcers often develop when reflux esophagitis complicates systemic sclerosis, but the prognosis is generally favorable.^9^ A case of a 63‐year‐old Japanese woman with long‐standing diffuse systemic sclerosis was further complicated by an aortoesophageal fistula associated with reflux esophagitis. The aortoesophageal fistula caused massive bleeding, resulting in her death.^11^

This case of a patient with systemic sclerosis who also presented with vesicovaginal and vesicorectal fistulas emphasizes this disease's complexity and multisystemic nature. Systemic sclerosis involves multiple organs, and its manifestations can be highly variable, making diagnosis and management challenging. It is crucial for clinicians to maintain a high index of suspicion for systemic sclerosis when encountering patients with diverse symptoms that do not improve with initial treatments.

The presence of vesicovaginal and vesicorectal fistulas in this patient with systemic sclerosis is a unique finding, as no previous cases have been reported in the literature. Fistulas, which are abnormal connections between two organs or vessels, can significantly impact the quality of life of affected patients, causing urinary and fecal incontinence, infection, and pain. The etiology of these fistulas in the context of systemic sclerosis is unclear and warrants further investigation.

Moreover, gastrointestinal involvement in systemic sclerosis is relatively common, with esophageal manifestations being the most frequent. The development of erosive esophagitis and abnormal esophageal motility in patients with systemic sclerosis can lead to complications, such as aortoesophageal fistulas, which can be life‐threatening.^12^ This highlights the importance of early identification and management of gastrointestinal complications in patients with systemic sclerosis.

This case serves as a reminder that systemic sclerosis is a complex disease with diverse presentations and potential complications. Clinicians should be prepared to consider a wide range of manifestations and complications when evaluating and managing patients with systemic sclerosis. Furthermore, this case encourages further research into the potential association between systemic sclerosis and the development of fistulas. Understanding this relationship could lead to better prediction, treatment, and prevention of complications associated with both conditions in the future.

We justify the link between systemic sclerosis and the presence of vesicovaginal and vesicorectal fistulas by presenting a comprehensive overview of the patient's medical history and the complex manifestations of systemic sclerosis. We emphasize that systemic sclerosis is a connective tissue disease known for causing fibrosis in the skin and internal organs. The disease can manifest in multiple ways, affecting various organ systems, including the respiratory, gastrointestinal, skin, and renal systems. Respiratory complications, such as interstitial lung disease and pulmonary hypertension, are well‐documented in systemic sclerosis, as are gastrointestinal symptoms, which can include esophageal issues like heartburn and dysphagia. We provide a detailed account of the patient's clinical presentation, highlighting her systemic sclerosis diagnosis and the presence of vesicovaginal and vesicorectal fistulas. We underscore that gastrointestinal involvement is observed in a significant percentage of individuals with systemic sclerosis, and esophageal complications are common. We refer to studies that show that individuals with systemic sclerosis often develop conditions like erosive esophagitis, which can lead to severe complications like aortoesophageal fistulas.

In this context, we emphasize that the presence of vesicovaginal and vesicorectal fistulas in a patient with systemic sclerosis is a unique finding, as there have been no prior reported cases. We recognize the significance of this observation and the need for further research to investigate the etiology of these fistulas in the context of systemic sclerosis. By providing a thorough explanation of the patient's medical history, connecting it to known systemic sclerosis complications, and highlighting the rarity of her condition, we rationalize that this is not a random coincidence. Instead, we suggest that there may be an association between systemic sclerosis and the development of fistulas, thereby encouraging further investigation into this relationship.

## 5 LIMITATIONS

The authors of this case report acknowledge the unique and multifaceted nature of the patient's condition, which combines systemic sclerosis, vesicovaginal and vesicorectal fistulas, and a myriad of other associated health issues. They recognize the importance of exploring potential links between systemic sclerosis and fistula development, as this could lead to better diagnosis and treatment strategies for such complications. The report underscores the complexity of systemic sclerosis, which affects multiple organ systems and presents with highly variable manifestations, posing a diagnostic and management challenge. The authors emphasize the need for clinicians to maintain a high index of suspicion when encountering patients with diverse and treatment‐resistant symptoms. Furthermore, they highlight the significance of early identification and management of gastrointestinal complications in systemic sclerosis patients, as these complications can be life‐threatening.

In light of the patient's unique presentation with vesicovaginal and vesicorectal fistulas, the authors acknowledge the scarcity of reported cases in the literature and the need for further investigation into the etiology of such fistulas in the context of systemic sclerosis. This case serves as a reminder that systemic sclerosis is a complex disease with a range of presentations and potential complications, requiring clinicians to consider a broad spectrum of manifestations during evaluation and management. The authors emphasize the importance of future research to elucidate the underlying mechanisms of fistula development in systemic sclerosis and to develop better strategies for prevention and treatment. Ultimately, understanding the relationship between these conditions can significantly improve patient outcomes and the overall management of systemic sclerosis and its associated complications.

## AUTHOR CONTRIBUTIONS

**Mohammad Quteineh:** Supervision; validation; writing – original draft; writing – review and editing. **Sajedah N. Obeid:** Investigation; writing – original draft; writing – review and editing. **Khayry Al‐Shami:** Data curation; investigation; resources; validation; writing – original draft; writing – review and editing. **Hamdah Hanifa:** Writing – original draft; writing – review and editing.

## FUNDING INFORMATION

The authors received no specific funding for this work.

## CONFLICT OF INTEREST STATEMENT

The authors report no conflict of interest.

## CONSENT

Written informed consent was obtained from the patient to publish this report in accordance with the journal's patient consent policy.

## Data Availability

The data that support the findings of this study will be available on request from the Editor‐in‐Chief.

## References

[1] Denton CP , Khanna D . Systemic sclerosis. Lancet. 2017;390(10103):1685‐1699. 28413064 10.1016/S0140-6736(17)30933-9

[2] Bossini‐Castillo L , López‐Isac E , Mayes MD , Martín J , eds. Genetics of systemic sclerosis. Semin Immunopathol. 2015;37:443‐451. 26032405 10.1007/s00281-015-0499-z

[3] Perelas A , Arrossi AV , Highland KB . Pulmonary manifestations of systemic sclerosis and mixed connective tissue disease. Clin Chest Med. 2019;40(3):501‐518. 31376887 10.1016/j.ccm.2019.05.001

[4] Sobolewski P , Maślińska M , Wieczorek M , et al. Systemic sclerosis–multidisciplinary disease: clinical features and treatment. Reumatologia. 2019;57(4):221‐233. 31548749 10.5114/reum.2019.87619 PMC6753596

[5] Kröner PT , Tolaymat OA , Bowman AW , Abril A , Lacy BE . Gastrointestinal manifestations of rheumatological diseases. Am J Gastroenterol. 2019;114(9):1441‐1454. 31205138 10.14309/ajg.0000000000000260

[6] Czirjak L , Foeldvari I , Müller‐Ladner U . Skin involvement in systemic sclerosis. Rheumatology. 2008;47(suppl_5):v44‐v45. 18784142 10.1093/rheumatology/ken309

[7] Bose N , Chiesa‐Vottero A , Chatterjee S . Scleroderma renal crisis. Semin Arthritis Rheum. 2015;44:687‐694. 25613774 10.1016/j.semarthrit.2014.12.001

[8] Van Den Hoogen F , Khanna D , Fransen J , et al. 2013 classification criteria for systemic sclerosis: an American College of Rheumatology/European League against rheumatism collaborative initiative. Arthritis Rheum. 2013;65(11):2737‐2747. 24122180 10.1002/art.38098 PMC3930146

[9] Zamost BJ , Hirschberg J , Ippoliti AF , Furst DE , Clements PJ , Weinstein WM . Esophagitis in scleroderma: prevalence and risk factors. Gastroenterology. 1987;92(2):421‐428. 3491774 10.1016/0016-5085(87)90137-5

[10] Rose S , Young MA , Reynolds JC . Gastrointestinal manifestations of scleroderma. Gastroenterol Clin N Am. 1998;27(3):563‐594. 10.1016/s0889-8553(05)70021-2 9891698

[11] Kotani T , Takeuchi T , Makino S , et al. A fatal aorto‐oesophageal fistula complicating systemic sclerosis. Scand J Rheumatol. 2008;37(3):234‐235. 18465462 10.1080/03009740701867364

[12] Roberts H , Curnow PA . Unilateral limited scleroderma‐like changes following formation of an arteriovenous fistula. Australas J Dermatol. 2007;48(1):37‐39. 17222301 10.1111/j.1440-0960.2007.00325.x

